# Development and Characterization of Gelled Double Emulsions Based on Chia (*Salvia hispanica* L.) Mucilage Mixed with Different Biopolymers and Loaded with Green Tea Extract (*Camellia sinensis*)

**DOI:** 10.3390/foods8120677

**Published:** 2019-12-13

**Authors:** Diana A. Guzmán-Díaz, Mayra Z. Treviño-Garza, Beatriz A. Rodríguez-Romero, Claudia T. Gallardo-Rivera, Carlos Abel Amaya-Guerra, Juan G. Báez-González

**Affiliations:** 1 Universidad Autónoma de Nuevo León, Facultad de Ciencias Biológicas, Departamento de Alimentos, Av. Pedro de Alba s/n, Cd. Universitaria, C.P. 66455 San Nicolás de los Garza, NL, Mexico; lic.nut.aleguzman@gmail.com (D.A.G.-D.); mayra_trevinogarza@hotmail.com (M.Z.T.-G.); claudia.gallardorv@uanl.edu.mx (C.T.G.-R.); carlos.amayagr@uanl.edu.mx (C.A.A.-G.); 2 Universidad Autónoma de Nuevo León, Facultad de Agronomía, Francisco I. Madero S/N, Ex Hacienda el Cañada, 66050 Cd. Gral. Escobedo, NL, Mexico; rodriguez_beatriz@outlook.com

**Keywords:** gelled double emulsion, chia mucilage, green tea extract, antioxidant activity, stability

## Abstract

The aim of this research was to develop and characterize five gelled double emulsions based on chia mucilage (CM) and different biopolymers (κ-carrageenan, C; locust bean gum, L; thixogum, T; and whey protein concentrate, W) loaded with green tea extract (GTE). Gelled double emulsions consisted of W_1_ (whey-protein-concentrate/sodium-azide/NaCl/GTE)/O and (PGPR/canola-oi)/W_2_ (CM, CMC, CML, CMT and CMW), and were characterized based on physicochemical properties during 35 days of storage. Optical microscopy clearly showed the drops of the internal phase surrounded by droplets of oil dispersed in the second aqueous phase; the droplet size was higher for CMT and lowest for CMW. In addition, all emulsions were highly stable at creaming and were effective in reducing the loss of antioxidant activity (88.82%) and total phenols (64.26%) during storage; CMT, CML and CM were the most effective. Furthermore, all emulsions showed a protective effect by modulating the release of the GTE in a simulated gastrointestinal environment, allowing a controlled release during the gastric-intestinal digestion phases and reaching its maximum release in the intestinal phase (64.57–83.31%). Thus, gelled double emulsions are an alternative for the preservation of GTE and could be a potential alternative for their application in the development of functional foods.

## 1. Introduction

Nowadays, consumers are focusing their attention on the consumption of high-quality foods that provide basic nutritional properties and provide beneficial effects to health, reducing the risk of disease [1,2]. Green tea is obtained from the *Camellia sinensis* L. plant native to China and is one of the most popular and beverages consumed around the world [3,4]. Green tea extract (GTE) is mainly composed of polyphenols, such as catechins (e.g., (−) epigallocatechin gallate, (−) epicatechin gallate, (−) epillogallocatechin and (−) epicatechin), gallic acid, quercitin and caffeine, among other substances [5,6]. Due to its composition, green tea has a wide variety of antioxidant, antimicrobial, anticancer, anticardiovascular and antihyperglycemic properties, among others [2,7,8]. In recent years, interest in the development of a wide variety of food products (e.g., meat, lactic and bakery products) supplemented with GTE has increased [6,9,10]. However, the problem associated with the incorporation of green tea into various food systems is related to the stability of biologically active compounds during processing and conservation. Factors such as oxygen, temperature, pH and added ingredients, among others, contribute to the degradation of active compounds, mainly of catechins, limiting their bioavailability [5,11]. Among the strategies used to reduce the degradation of GTE, those which stand out are the use of techniques such as spray chilling [12], spray-drying [13], freeze drying [14], electrospraying [15], liposomes [16], high sheer homogenization and high pressure homogenization, such as nanoparticles [17], nanoemulsions [18], film formation [19], simple [20] and double emulsions [21,22].

Double emulsions (W_1_/O/W_2_) are systems consisting of an internal aqueous phase (W_1_), trapped as small drops within larger oil droplets (O), which are subsequently dispersed in another aqueous phase (W_2_) [23,24,25]. Double emulsions encapsulating GTE have been developed for cosmetic use and food, among other applications. However, the use of these systems has been limited due to long-term physical destabilization processes, manifested by phenomena such as creaming, phase inversion or phase separation, as well as flocculation and coalescence. It has been shown that biopolymers can form a gel network in the continuous phase improving the stability of multiple emulsions during storage time [26,27]. There are studies of double emulsions (W_1_/O/W_2_) based on extra pure paraffin oil/cetyl dimethicone copolyol/polysorbate 80) incorporated with GTE (5%). However, although such emulsions were stable enough against any phase separation, some parameters, such as pH, viscosity and conductivity, were not impressive enough to assume multiple emulsions in the long term (30 days) [21]. In another study, Mahmood et al. [22] evaluated the stability of double emulsions (W_1_/O/W_2_) based on cetyl dimethicone copolyol (lipophilic emulsifier) and a blend of polyoxyethylene (20) cetyl ether and cetomacrogol 1000^®^ (hydrophilic emulsifiers) with the presence of a thickener and hydroxypropyl methylcellulose (HPMC); these thickeners considerably improved the stability of W_1_/O/W_2_ for a period of 12 months.

On the other hand, chia (*Salvia hispanica* L.) is an herbaceous plant originally from southern Mexico. Chia seed consumption is related with beneficial effects on health due to its high levels of protein, minerals, vitamins, antioxidants and dietary fiber (5–6% of mucilage) [28,29,30]. Chia mucilage (CM) is an anionic heteropolysaccharide consisting of a tetrasaccharide with 4-O-methyl-α-D-glucoronopyranosyl residues occurring as branches of β-D-xylopyranosyl on the main chain [31]. CM is a promising alternative as an ingredient in the food industry due to its high fiber content, excellent water retention capacity, high solubility and viscosity at low concentrations, among other properties [28,29]. Recent research has focused on the use of CM as a biomaterial for stabilizing emulsions for human consumption [32]. In addition, Capitani et al. [28] found that the incorporation of CM to simple emulsions (O/W) increased their stability against coalescence and the gravitational phase separation by increasing the viscosity of the aqueous phase, limiting the mobility of the oil droplets in the emulsions. Nonetheless, although there are some studies on the effect of CM in the stability of emulsions, to our knowledge, there have been no previous studies with respect to gelled double emulsions incorporated with GTE. Thus, the aim of this research was to develop and characterize gelled double emulsions based on CM mixed with different biopolymers and loaded with GTE as a function of storage time.

## 2. Materials and Methods

### 2.1. Materials

GTE was obtained from Organic by the Cup S.A. de C.V. Canola oil and chia seed were purchased from a local supermarket in Monterrey, Nuevo León, México. Thixogum^TM^ (T; a synergistic co-processed blend of highly purified gum acacia and xanthan), locust bean gum (L), and κ-carrageenan (C) were acquired from Gomas Naturales, S.A. de C.V. (Ciudad de México, México). Whey protein concentrate (W) was purchased from Ingresa México S.A. de C.V. (Monterrey, Nuevo León, México). Polyglycerol polyricinoleate (PGPR 4125, HLB ~1.5) was donated by Millikan, S.A de C.V (Tlalnepantla, Estado de México, Mexico). Free radicals; 1,1-diphenyl-2-picrylhdrazyl (DPPH+), 2,2′-Azino-bis(3-ethylbenzthiazoline-6-sulfonic acid) (ABTS+) and 6-hydroxy-2,5,7,8-tetramethylchroman-2 carboxylic acid (Trolox) were obtained from Sigma-Aldrich Química, S. de R. L. de C.V. (Toluca, Estado de México, México). Ethanol, *n*-hexane, acetone, sodium azide and sodium chloride were purchased from Desarrollo de Especialidades Químicas S.A de C.V (Monterrey, Nuevo León, México).

### 2.2. Obtention of Green Tea Extract (GTE)

GTE was extracted by constant magnetic stirring (Thermo Scientific, Super Nuova, Inc., Waltham, MA, USA) with an ethanolic solution (80%) at a constant ratio 1:100 (w/v) for 4 h. Subsequently, the solution was subjected to an ultrasonic bath (conditions), filtered under vacuum (Whatman filter paper No.1), and ethanol was evaporated in a rotary evaporator (Rotavapor Lauda Alpha, IKA Instruments, Wilmington, NC, USA). The concentrated solution was kept frozen with CO_2_ and then freeze-dried for 24 h [33]. Yield was determined according the following equation:

Yield (%) = ((mass of GTE)/(mass of green tea)) × 100.(1)

#### 2.2.1. Characterization of GTE

• Antioxidant Activity and Total Phenol Content

Antioxidant activity of GTE (500 mg kg^−1^) was measured using the DPPH+ and ABTS+ methods during 35 days of storage (0, 7, 14, 21, 28 and 35 days). Trolox was used as a standard reference. A DPPH+ radical scavenging assay was determined following the method of Thaipong et al. [34], with some modifications; a DPPH+ solution (0.1 mM) was prepared in ethanol at 96% and then was measured in a spectrophotometer (Genesys 5, Thermo Spectronic, Rochester, NY, USA) at 517 nm (absorbance = 1.00 ± 0.02). Aliquots of GTE (600 μL) were mixed with 2.27 mL of DPPH+. The mixture was then allowed to react for 2 h at 25 ± 2 °C in dark conditions and the absorbance was recorded at 517 nm. A standard calibration curve of Trolox was determined for the DPPH+ radical at concentrations ranging from 0 to 300 μmol. The curve equation was y = 0.7736x + 3.6801, R^2^ = 0.99. Antioxidant activity values were expressed as µmol Trolox equivalent (TE) mL^−1^.

Moreover, an ABTS+ radical scavenging assay was measured according to Jin et al. [4] with some modifications. An ABTS+ solution was prepared in ethanol 96° (7.0 mmol L^−1^) and allowed to react with a potassium persulfate solution (2.45 mmol L^−1^) at a 1:1 ratio (v/v) for 16 h at 25 ± 2 °C in dark conditions, the absorbance then being recorded by a spectrophotometer at 734 nm (absorbance = 0.700 ± 0.02). Aliquots of GTE (150 μL) were mixed with ABTS+ solution (2.7 mL), the mixture was allowed to react for 6 min at 25 ± 2 °C in dark conditions and the absorbance was recorded at 734 nm. Finally, a standard calibration curve of Trolox was determined for the ABTS+ radical at concentrations ranging from 0 to 300 μmol. The curve equation was y = 0.4387x + 3.7833, R^2^ = 0.99. Antioxidant activity values were expressed as µmol TE mL^−1^. Additionally, antioxidant activity for both the DPPH+ and ABTS+ methods was calculated as a percentage of inhibition according to the following equation:

Inhibition (%) = ((Absorbance of control without GTE) − (Absorbance of sample))/(Absorbance of control without GTE) × 100.(2)

Finally, total phenol content was determined by the Folin–Ciocalteu method as reported by Thaipong et al. [34], with some modifications. Briefly, GTE (600 μL), distilled water (1 mL) and Folin–Ciocalteu reagent (1N; 100 μL) were mixed (3 min) and a solution of Na_3_CO_2_ (20%; 300 μL) was subsequently added and allowed to react for 90 min. Absorbance was measured at 734 nm. A standard calibration curve was determined for catechin at concentrations range of 20–180 μmol L^−1^. The curve equation was y = 0.0037x − 0.0162, R^2^ = 0.99. Total phenol content was expressed as µg catechin equivalents (CE) mL^−1^_._

### 2.3. Extraction of Chia Mucilage

CM extraction was carried out according to the methods reported by Capitani et al. [28] and Timilsena et al. [35] with some modifications. Chia seeds (100 g) were washed with ethanol (200 mL) under magnetic stirring (2 min) to remove impurities and were subsequently recovered with a strainer. Seeds were placed in distilled water (1:1000 p/v) and kept under constant magnetic stirring for 4 h. Hydrated seeds were freeze-dried for 5 days and CM was separated from seeds using sieves (mesh; 297 μm). The CM recovered (off-white powder) was stored in plastic containers until later use. Yield was determined according the following equation:

Yield (%) = ((mass of chia seeds)/(mass of chia mucilage)) × 100.(3)

### 2.4. Preparation of Gelled Double Emulsions

The inner aqueous phase (W_1_) was prepared dissolving WPC, sodium azide, NaCl and GTE (500 mg kg^−1^ to obtain a final concentration of 100 mg kg^−1^) into distilled water by constant magnetic stirring (2 h), and the oil phase (O) was prepared by dispersing PGPR into canola oil at 50 °C to reduce the viscosity of these solutions. The W_1_ phase (ф_1_ = 0.2; weight fraction of the dispersed phase) was dispersed into the O phase with a high-power homogenizer (Ultra Turrax IKA-T50 digital, Werke, Staufen, Germany) at 7500 rpm for 5 min^−1^ (primary emulsion). Five different solutions for the external aqueous phases (W_2_) were prepared; CM (alone) and in combinations (1:1), CMC (chia mucilage/κ-carrageenan), CML (chia mucilage/locust bean gum), CMT (chia mucilage/thixogum) and CMW (chia mucilage/whey protein concentrate) by constant magnetic stirring until completely dissolved; concentrations were established based on previous experiments (data not shown). Finally, the W_1_O phase was dripped into the W_2_ phase (ф_2_ = 0.2), followed by mixing with a high-power homogenizer at 7500 rpm for 5 min^−1^ (double emulsion, W_1_/O/W_2_). Finally, gelled double emulsions obtained were stored at 4 °C for 35 days. The compositions of the gelled double emulsions are shown in Table 1.

#### 2.4.1. Characterization of Gelled Double Emulsions

• Optical Microscopy

Microscopic images of gelled double emulsions (day 0 of storage) were obtained using a conventional optical microscope (Leica DM 500, Leica Microsystems, Wetzlar, Germany) equipped with a camera and Image pro plus 4.0 software. The emulsions were observed with a 100× magnification objective, using immersion oil.

• Droplet Size Determinations

Droplet size measurements of the gelled double emulsions were made using static light scattering with Mastersizer 3000 equipment (Malvern Instruments, Malvern, UK). Results were expressed as the mean droplet size (D_4,3_ and D_3,2_ µm). Measurements were made at days 0, 7, 14, 21, 28 and 35 of storage, respectively.

• Creaming Index

Creaming stability measurements of gelled double emulsions were performed according to Surh et al. [23] with slight modifications. Emulsions (10 g) were transferred into a plastic container (15 mL) and stored at 4 °C for 35 days. Creaming stability was determined according the following equation:

Creaming index (%) = ((total height of the emulsion)/(height of serum layer)) × 100.(4)

• Antioxidant Activity and Total Phenolic Content

The protective effect of gelled double emulsions was determined by evaluating the antioxidant activity and total phenolic content of GTE loaded into the emulsions during 35 days of storage. Firstly, the breaking of the gelled double emulsions was carried out as reported by Rodríguez-Huezo et al. [36] with some modifications. Emulsions (1 g) were collocated separately in a centrifuge bottle containing 3 mL of a solution of NaCl (10%)/methanol (1:1) and stirred for 10 min. Then 8 mL of a solution of hexane/acetone (1:1) were added and the mixture remained in constant agitation in a vortex (2 min; Mixer Labnet Internacional, Inc, Woodbridge, NJ, USA), subsequently placed in an ultrasonic bath (70 Hz; Sonic Ruptor 250, Omni International, Inc., Marietta, GA, USA) for 10 min. Finally, the mixture was centrifugated (10,000 rpm for 30 min) and the mean organic fraction resulting from this process was used for antioxidant activity and total phenols determinations (Section 2.2.1). The protective effect of gelled double emulsions was determined at days 7, 14, 21, 28 and 35 of storage, respectively. Results were expressed as both loss of antioxidant activity (DPPH+ and ABTS+) and loss of total phenolic content according to the following equation:

Activity loss (%) = ((measurements at different days)/(measurements at day 0)) × 100.(5)

• Bioavailability (In Vitro Digestion Process)

In vitro digestion processes were conducted as reported by Aceituno-Medina et al. [37] with some modifications. For the gastric digestion phase, separately, solutions containing gelled double emulsions and distilled water were prepared (30 mL in a ratio 1:1). These solutions were acidified with HCl (6 M) to obtain a pH = 2 and subsequently were added into a porcine pepsin solution (0.6 mL; 160 mg mL^−1^ previously prepared in a solution of HCl 0.1 M) and distilled water to obtain a final volume of 40 mL. This solution remained under constant stirring for 2 h at 37 °C in dark conditions (0–120 min). After gastric digestion, the pH of the solution was adjusted to 7 by addition of NaHCO_3_ (0.045 M) and a pancreatin-bile solution (2.4 mL; pancreatin 4 mg mL^−1^, bile 25 mg mL^−1^ prepared in 0.1 NaHCO_3_) was added; the mixture was stirred at 37 °C for 2 h in dark conditions (intestinal digestion phase; 120–240 min). GTE released throughout gastric (10, 30, 60, 90 and 120 min, respectively) and intestinal (130, 160, 180, 210 and 240 min, respectively) conditions was calculated by measuring the samples (600 μL) obtained directly from the gastrointestinal medium at different times, by the antioxidant activity method (ABTS+) as reported in a previous section (Section 2.2.1). GTE released was calculated according the following equation:

GTE released (%) = ((measurements at different times)/(measurements at 0 min)) × 100.(6)

Finally, parameters such as mean droplet size D_4,3_ (µm) and microscopic images of the gelled double emulsions during the in vitro digestions process were obtained as reported in a previous section (Section 2.4.1).

### 2.5. Statistical Analysis

All determinations were carried out in triplicate (*n* = 3). Statistical analysis of the data was performed using SPSS software (IBM version 22, SPSS Inc, Chicago, IL, USA). Data were analyzed through a variance test (ANOVA) and a Tukey test. Differences between means were considered significant at *p* values ≤ 0.05.

## 3. Results and Discussion

### 3.1. Obtention of Green Tea Extract (GTE)

The GTE had a yield of 28.2% total dry matter (data no shown), similar to the reported by Unno and Osakabe [38].

#### Antioxidant Activity and Total Phenolic Content of GTE

**Figure 1:**
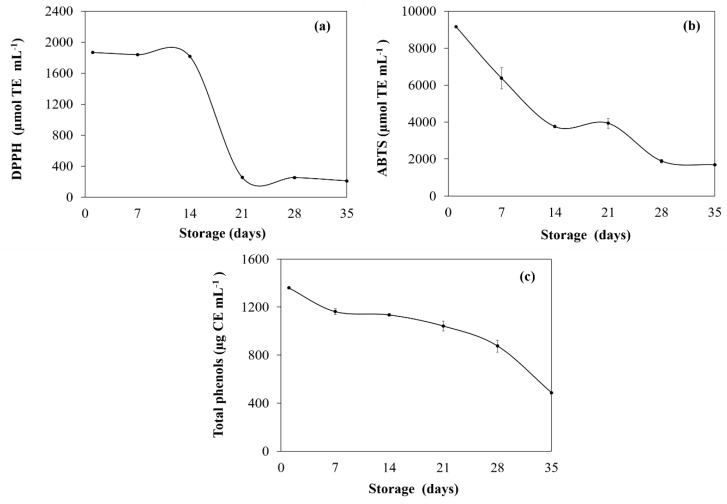
Antioxidant properties—(**a**) DPPH+ radical scavenging assay, (**b**) ABTS+ radical scavenging assay, and (**c**) total phenolic contents—of green tea extract (GTE) stored at 4 °C during 35 days of storage. Mean values ± standard deviation (*n* = 3).

Results obtained regarding antioxidant activity and total polyphenol content are shown in Figure 1. The measurement of antioxidant activity performed by the DPPH+ method ranged between 1868.57 and 208.86 μmol TE mL^−1^ (% inhibition: 53.53% and 9.25% at days 0 and 35, respectively; data not shown) and for ABTS+ between 9164.26 and 1685.45 μmol TE mL^−1^ (% inhibition: 84.19% and 18.57% at days 0 and 35, respectively; data not shown). Similar results have been reported by Wang and Liang [39] and Afify et al. [40]. In both methods, the same behavior was observed with a significant loss of antioxidant activity at the end of storage time (*p* < 0.05; 88.82% and 81.60% for DPPH+ and ABTS+, respectively). On the other hand, GTE showed an initial total phenols content of 1360.49 μg CE mL^−1^, with a significant loss of up to 64.26% (486.14 μg CE mL^−1^) for day 35 of storage. Higher values of this parameter have been reported in previous studies [41,42]. This effect may be associated with the extraction method. According to the report by Friedman et al. [43], the loss of antioxidant activity and polyphenols in GTE over time can be attributed to a progressive degradation of catechins levels, mainly of the -epigallocatechin and -epigallocatechin 3-gallate.

### 3.2. Extraction of Chia Mucilage (CM)

CM yields obtained in this study were of 5.31% total dry matter (data not shown), similar to that found by Campos et al. [44]. In addition, higher values were reported by Muñoz et al. [45] with yields of 6.97%; this difference can be linked to the seed variety and to the extraction method.

### 3.3. Characterization and Stability of Gelled Double Emulsions

#### 3.3.1. Microscopic Analysis

**Figure 2:**
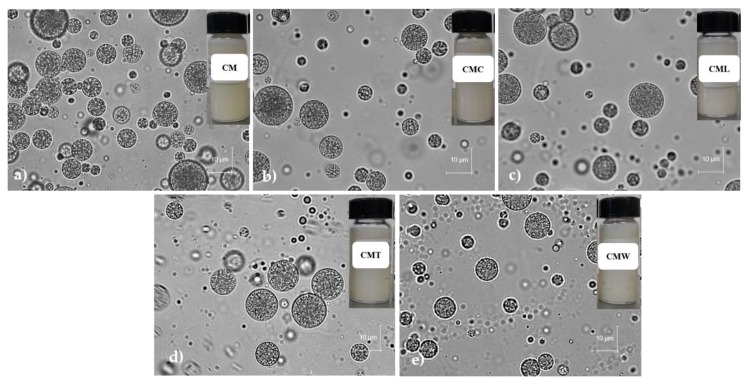
Microscopic images of the gelled double emulsions—(**a**) CM, (**b**) CMC, (**c**) CML, (**d**) CMT y (**e**) CMW—at day 0 of storage. Note: CM = chia mucilage with the different biopolymers (κ-carrageenan, C; locust bean gum, L; thixogum, T; and whey protein concentrate, W).

Gelled double emulsions based on CM and mixtures with different biopolymers (CMC, CML, CMT and CMW) encapsulating GTE were obtained. In Figure 2 the microscopic images of the gelled double emulsions at days 0 of storage are shown. In general, in all emulsions the drops of the internal phase surrounded by oil droplets dispersed in the second aqueous phase were clearly observed, which are typical characteristics of double emulsions [23,46,47].

#### 3.3.2. Droplet Size Measurements

**Figure 3:**
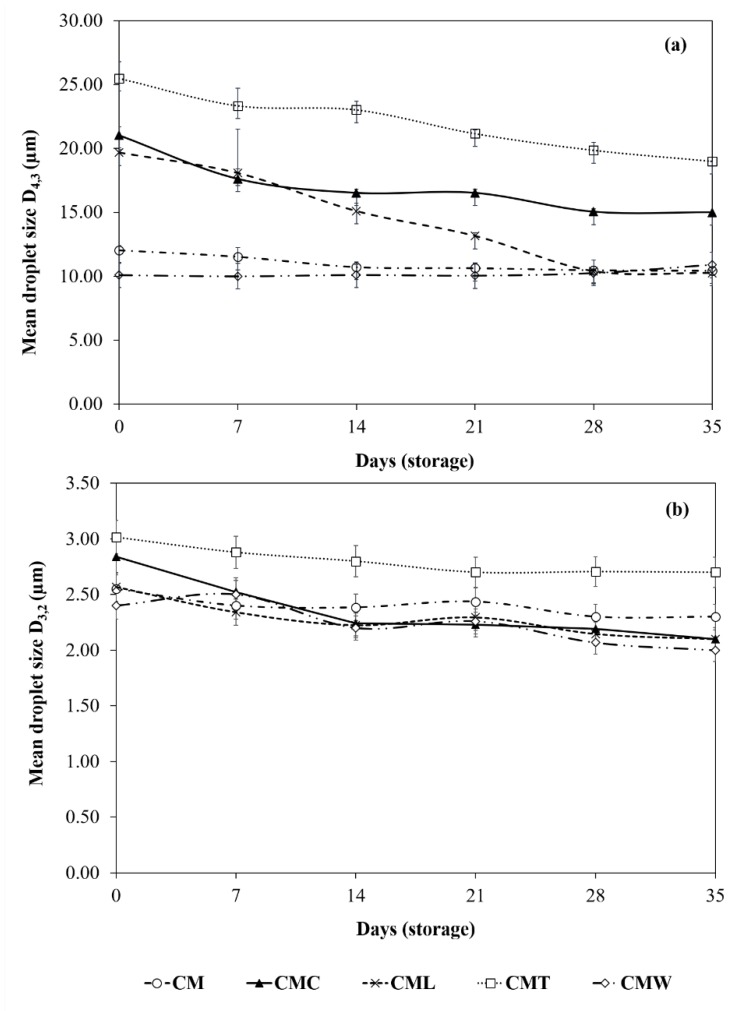
Mean droplet size—(**a**) D_4,3_ and (**b**) D_3,2_—of the gelled double emulsions stored at 4 °C during 35 days of storage. Mean values ± standard deviation (*n* = 3).

The droplet size was significantly different (*p* < 0.05) between the gelled double emulsions (day 0), the highest value was presented by CMT (D_3,2_ = 3.02 µm and D_4,3_ = 25.48 µm) and the lowest was for CMW with values of D_3,2_ = 2.42 µm and D_4,3_ = 10.10 µm. Intermediate sizes were presented by CM, CML and CMC (D_3,2_ = 2.55–2.84 µm and D_4,3_ = 12.02–21.04 µm; Figure 3). According to Mohammadi et al. [26], the simultaneous use of two biopolymers can increase the thickness of the emulsifier layer around the droplets and therefore increase the droplet size, similar to that found in CML, CMC and CMT. On the other hand, Capitani et al. [28] report that this effect can also be associated with an increase in the viscosity of the aqueous phase generated by the different biopolymers, since the viscosity could affect the homogenization process, preventing the complete disruption of the droplets and resulting in a larger population of droplets, similar to that found in CML, CM, CMT and CMC gelled double emulsions (viscosity = 10.15–104.53 Pa·s at a shear rate γ = 0.1 seg^−1^, data not shown). However, this behavior was not found in CMW because the WPC forms a weak gel of low viscosity (viscosity = 2.23 ± 0.43 Pa·s at a shear rate γ = 0.1 seg^−1^, data not shown). Compared to the literature, particle size values lower than CML, CMC and CMT [21,48] and similar to CMW and CM have been reported in previous studies of double emulsions encapsulating green tea [21]. On the other hand, regarding the particle size during storage, CMW and CM emulsions remained significantly stable (*p* < 0.05), with values of 10.90 µm and 10.44 µm (D_4,3_), respectively, for day 35 of storage. The lower droplet size values found in CMW gelled double emulsion during storage can be attributed to the fact that CMW contains a significant amount of proteins, which are surface active, and have better emulsifying properties, resulting in a more stable emulsion [20]. Similarly, CM (alone) is active on the surface since it forms small droplets that result in a stable emulsion [28]. Moreover, for emulsions CMC, CML and CMT, a decrease (*p* < 0.05) in the particle size was observed from day 7 of storage, with final values of 15.00, 10.26 and 19.00 µm (D_4,3_), respectively, in accordance with previous reports [49]. This effect could be associated with the fact that these mixtures are not particularly active on the surface, leading to emulsions with larger droplet sizes. In general, this behavior was also found in D_3,2_ values throughout the storage period. This droplet size reduction could be associated with a leakage of water from the internal aqueous phase to the external phase of the emulsions; similar behavior has been previously reported in double nanoemulsions loaded with green tea [22].

#### 3.3.3. Creaming Stability Measurements

Gelled double emulsions were highly stable against creaming during 35 days of storage (creaming index = 0%), indicating that CM and its simultaneous use with CMC, CML, CMT and CMW were an adequate alternative to stabilize the external drops in the double emulsions due to the fact that they form a thick polymeric network of strong gelation that improves the mechanical and steric stability, providing resistance to the rupture of the drops (elastic modulus (G’) of CM = 80.792 Pa, CMW = 13.463 Pa, CML = 69.020 Pa, CMT = 98.109 Pa and CMC = 564.428 Pa, at a shear rate γ = 0.1 seg^−1^, data not shown [28,50,51]). Finally, our results are in agreement with Ye et al. [52], who reported that the incorporation of polysaccharides in the external aqueous phase at concentrations >0.1% avoid creaming during storage time.

#### 3.3.4. Protective Effect of Gelled Double Emulsion on GTE

**Figure 4:**
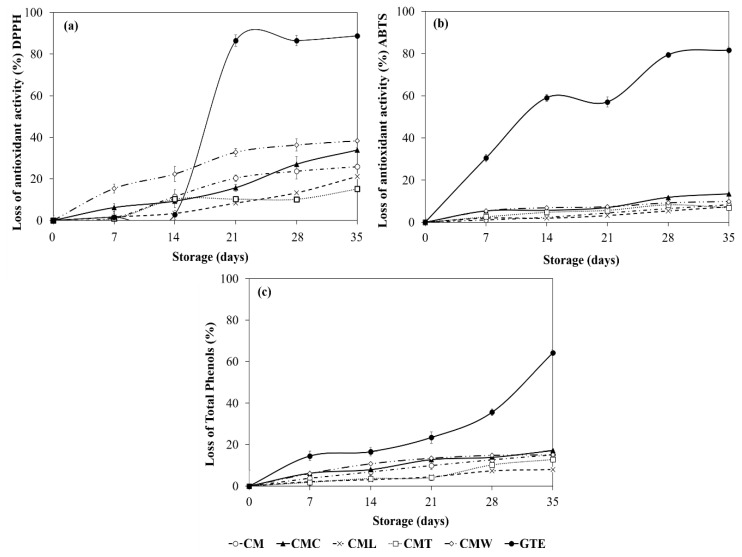
Antioxidant properties—(**a**) DPPH+ radical scavenging assay, (**b**) ABTS+ radical scavenging assay, and (**c**) total phenolic contents—of gelled double emulsions and GTE stored at 4 °C during 35 days of storage. Mean values ± standard deviation (*n* = 3).

The protective effect against GTE degradation during storage by DPPH+, ABTS+ and the total phenolic content is shown in Table 2 and Figure 4. Initial antioxidant activity values performed by the DPPH+ and ABTS+ methods ranged between 1954.50 and 2333.15 μmol TE mL^−1^, and between 17,343.51 and 19,098.70 μmol TE mL^−1^ for gelled double emulsions, respectively. Regarding total phenolic content, gelled double emulsions had values ranged from 2656.36 to 4135.97 μmol CE mL^−1^ (Table 2). In general, CML gelled double emulsion showed the higher values in these parameters. On the contrary, GTE showed the lowest values of antioxidant activity (DPPH+ = 1868.57 μmol TE mL^−1^ and ABTS+ = 9164.30 μmol TE mL^−1^) and total phenols (1360.49 μmol CE mL^−1^; day 0). Moreover, during storage time, in general, a significant decrease (*p* < 0.05) was observed in the antioxidant activity values in all treatments. However, this decrease was significantly higher (*p* < 0.05) in GTE, indicating that the gelled double emulsions had a protective effect against the degradation of antioxidants and phenolic compounds. By the end of storage (day 35), antioxidant activity values of gelled double emulsions ranged from 1361.83 to 1839.26 μmol TE mL^−1^ (DPPH+) and 15,898.77 to 17,689.77 μmol TE mL^−1^ (ABTS+), while the total phenols fluctuated between 2312.88 and 3805.70 μmol CE mL^−1^; again, the CML gelled double emulsion showed the highest values in these parameters (Table 2). Finally, GTE showed the lowest values of antioxidant activity and total phenols which were of 208.82 μmol TE mL^−1^ (DPPH+), 1685.45 μmol TE mL^−1^ (ABTS+) and 486.14 μmol CE mL^−1^, respectively (Table 2). In general, CMT, CML and CM gelled double emulsions presented a similar protection barrier in relation to the loss of antioxidant activity (day 35, 15.13–25.86% and 6.89–8.33% for DPPH+ and ABTS+, respectively) and polyphenols (7.99–15.13%) with respect to its initial values (day 0). In addition, although CMC showed high values of antioxidant activity and total phenols, this emulsion was less effective in preventing the degradation of antioxidants and polyphenols (DPPH+ = 33.48%, ABTS+ = 13.41% and total phenols = 17.25% at day 35) with respect to its initial values (day 0), compared to CMT, CML and CM. In the same way, although CMW showed high values of antioxidant activity and total phenols, this emulsion showed the highest values of loss of antioxidant activity (DPPH+ = 38.32% and ABTS+ = 9.82% at day 35) and polyphenols (15.12% at day 35) with respect to its initial values (day 0; Figure 4). Finally, GTE showed the highest loss of antioxidant activity (up to 88.82%) and of total phenols (64.26%) during storage (Figure 4). In general, gelled double emulsions stabilized with CM and combined with other polysaccharides, such as tixogum, locust bean gum and κ-carrageenan, were effective in reducing the loss of antioxidant activity and polyphenols throughout storage and this fact can be attributed to the fact that these polysaccharides act as thickening agents, increasing the viscosity of the external aqueous phase and forming a strong polymer network that traps the active compound within its structure, acting as a protective barrier against GTE degradation [24]. On the other hand, the loss of activity in CMW could be associated with the formation of a weaker polymer network (weak gels) [53,54]. It also has been reported that the protein–polyphenol interactions of the external phase could affect and mask the antioxidant capacity of the phenolic compounds [55,56].

#### 3.3.5. Digestibility (In Vitro) of Gelled Double Emulsions

• Bioavailability (In Vitro Digestion Process)

**Figure 5:**
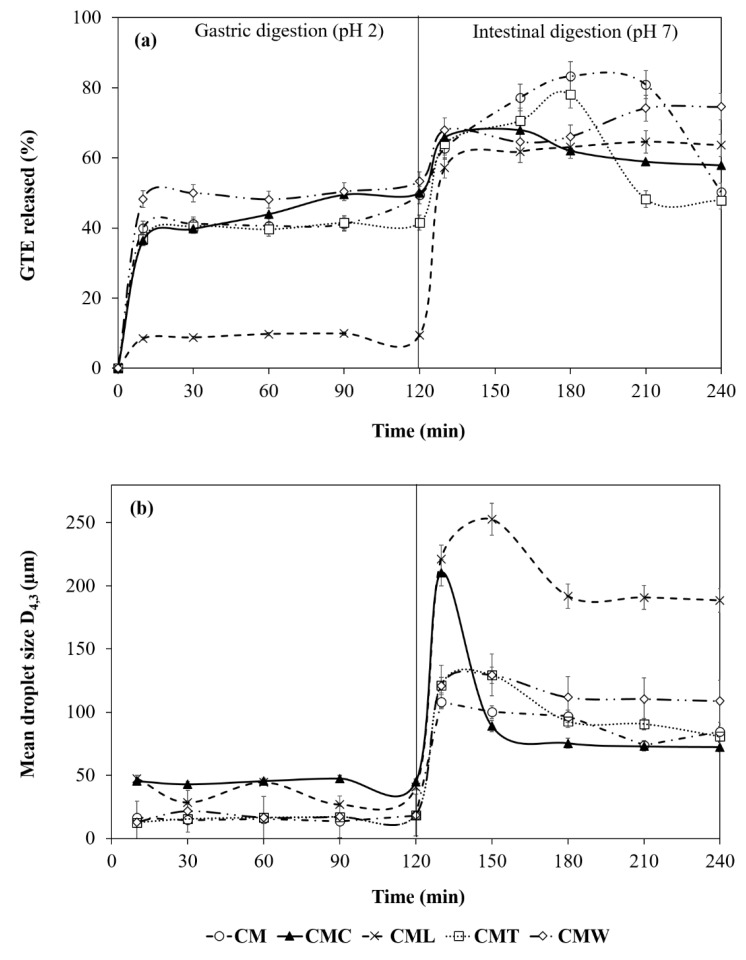
(**a**) Release profile of GTE, and (**b**) mean droplet diameter of gelled double emulsions—during the gastric and intestinal in vitro digestion process (day 0). Mean values ± standard deviation (*n* = 3).

The release profiles of the gelled double emulsions loaded with GTE (day 0) during the in vitro digestion process are shown in Figure 5. Between 0 and 10 min a significant increase (*p* < 0.05) in the release of GTE was observed in CMC, CMT, CM (36.47–39.94%), followed by CMW (48.22%) and CML (8.47%), whose values were lower compared to the rest of the treatments. In addition, between 30 and 120 min a significant stability (*p* < 0.05) was observed in the release profile in both CML, CMT, CM and CMW emulsions (9.42%, 41.55%, 49.42% and 53.33% at 120 min, respectively). The CMC double emulsion was the most sensitive to the process of gastric digestion, presenting a constant release between 10 and 120 min (36.47%–50.05%). As reported by Lamothe et al. [9] and Namal [6], the effect of stability observed in the in vitro gastric digestion process can be attributed to the fact that some phenolic compounds of the GTE (e.g., catechins and gallic acid) are stable at pH acids similar to those reported in the stomach (pH < 4). In addition, these polyphenols can interact with the pepsin, resulting in a change in their molecular conformation and reducing their catalytic activity. On the other hand, in the transition from the gastric to the intestinal phase a rapid increase (*p* < 0.05) in the release profile was again observed in all treatments. CMC, CM and CMT gelled double emulsions were more easily digested and presented a higher release percentage at 160 min (67.98%) and 180 min (83.31% and 78.06%), respectively. In addition, CML and CMW showed their greatest release at 210 and 240 min with values of 64.57% and 74.58%, respectively. This effect may be associated with the instability of GTE catechins at alkaline pH (>6). These findings indicate that the gelled double emulsions can act as a protective barrier by modulating the polyphenol release process in a simulated gastrointestinal environment, preserving its stability and allowing a controlled release of GTE in the small intestine [9,14,57]. Finally, antioxidant activity values of both gelled double emulsions and the GTE (free) throughout the in vitro digestion phases are shown in the following section.

• Antioxidant Activity during In Vitro Digestion Process

Regarding the antioxidant activity throughout the in vitro digestion phases, significant differences were found between the treatments and among the phases of the digestion process (*p* < 0.05; Table 3). Antioxidant activity values in the initial phase (10 min) were higher for GTE free (8984.24 µmol TE mL^−1^), followed by CMW (7315.70 µmol TE mL^−1^). On the other hand, CMT, CM and CMC presented similar values fluctuating between 5059.73 and 5265.95 µmol TE mL^−1^, while the lowest value was for CML (1466.48 µmol TE mL^−1^). Between 10 and 120 min of the gastric digestion phase, an increase in antioxidant activity was found for both free GTE and gelled double emulsions. At the end of this process, the antioxidant activity was higher for GTE (9315.45 µmol TE mL^−1^) because it is free, followed by CMW (8090.60 µmol TE mL^−1^), CMC (7228.21 µmol TE mL^−1^), CM (6353.32 µmol TE mL^−1^), CMT (5697.15 µmol TE mL^−1^) and CML (1631.46 µmol TE mL^−1^; Table 3). The high activity values of the GTE with respect to double gelled emulsions are attributed to a controlled release process, consistent with that found in the GTE release profile (Figure 5). Finally, the values of antioxidant activity at the end of intestinal digestion (240 min) were lower for CM and CMT (6465.80–6565.79 µmol TE mL^−1^), followed by CMC (8365.56 µmol TE mL^−1^), CML and CMW (11,008.98–11,315.20 µmol TE mL^−1^), similar to that found in the release profile (Figure 5), while the highest values were for free GTE (16,470.80 µmol TE g^−1^; Table 3).

• Droplet Size during In Vitro Digestion Process

On the other hand, in the simulated digestion process, only slight fluctuations in particle size were found during the gastric digestion phase in all treatments, in accordance with previous reports [58,59]. These slight fluctuations could be associated with the instability of double gelled emulsions due to the effect of acidic pH and pepsin. However, it has been reported that the addition of GTE significantly reduces the degradation of various food matrices during the gastric digestion process [9]. Furthermore, during the intestinal digestion process a significant increase (*p* < 0.05) was observed in the droplet size of CM (108.20 µm; 130 min), CMC (210.40 µm; 130 min), CMW (129.33 µm; 150 min), CMT (129.20 µm; 150 min) and CML (252.60 µm; 150 min), due to the effect of basic pH, bile salts and pancreatin [60], followed by a gradual decrease in the size of the drop between 150 and 240 min, associated with the release of the GTE in the intestinal phase and consistent with that found in the release profile of the GTE (Figure 5).

• Optical Microscopy during In Vitro Digestion Process

**Figure 6:**
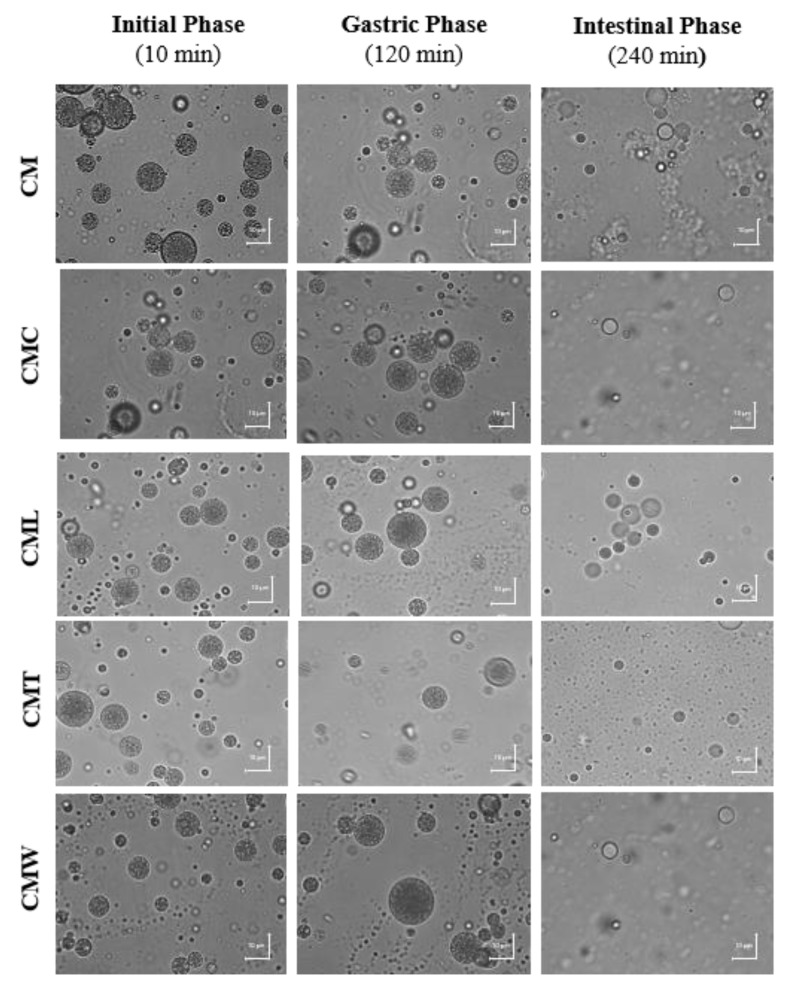
Microscopic images of the gelled double emulsions—before and after the gastric and intestinal in vitro digestion phases.

In the microscopy of the gelled double emulsions at the end of the gastric digestion phase (120 min), the drops of the internal phase surrounded by oil droplets dispersed in the second aqueous phase were observed, indicating that the emulsions maintained the typical morphology of a double emulsion during this process (Figure 6.). On the contrary, in microscopy of the emulsions after the intestinal digestion phase (240 min) internal drops were evidenced in smaller quantity and size, indicating a partial migration from the internal phase (W_1_) to the external phase (W_2_), associated with the release of the GTE [61].

## 4. Conclusions

GTE and CM were obtained with yields of 28.2% and 5.31%, respectively. GTE presented a high antioxidant activity (DPPH+ = 1868.57 and ABTS+ = 9164.30 µm TE mL^−1^) and a high total phenolic content (1360.49 μg CE g^−1^), with an activity loss of up to 88.82%, 81.60% and 64.26%, respectively, in such parameters after 35 days of storage. Moreover, five gelled double emulsions based on CM and mixtures with different biopolymers (CMC, CML, CMT and CMW) loaded with GTE were obtained. Optical microscopy of emulsions clearly showed the drops of the internal phase surrounded by droplets of oil dispersed in the second aqueous phase, which are typical characteristics of double emulsions. The droplet size of the gelled double emulsions fluctuated between 2.42 and 3.02 µm (D_3,2_) and 10.10 and 25.48 µm (D_4,3_), and the lowest values were for CMW while the highest values were for CMT. In general, all gelled double emulsions were highly stable at creaming and were effective in reducing the loss of antioxidant activity (up to 25.86% for DPPH+) and total phenols (15.13%) during 35 days of storage; CMT, CML and CM were the most effective. On the other hand, overall gelled double emulsions showed a protective effect by modulating the release of the GTE in a simulated gastrointestinal environment, allowing a controlled release during the gastric-intestinal digestion phases and reaching its maximum release in the intestinal phase (64.57–83.31%). Although evaluations of the in vitro digestion process provide important information on the behavior of double gelled emulsions and GTE, it is necessary to continue with this research in order to determine the effect of the gastrointestinal environment on the gelled double emulsions and GTE during different storage times. Thus, gelled double emulsions are an alternative to preserve GTE and could be a potential alternative for their application in the development of functional foods.

## Figures and Tables

**Table 1:** Chemical composition of gelled double emulsions.

Primary Emulsion (W_1_/O) (ф_1_ = 0.2)						Second Aqueous Phase (W_2_) (ф_2_ = 0.2)				
Aqueous Phase					Canola Oil Phase	Aqueous Phase				
Emul-sions	WPC (%)	NaCl (%)	Sodium azide (%)	GTE mg kg^−1^	PGPR (%)	CM (%)	C (%)	L (%)	T (%)	W (%)
CM	15	0.02	0.02	500	7.5	2	-	-	-	-
CMC	15	0.02	0.02	500	7.5	1	1	-	-	-
CML	15	0.02	0.02	500	7.5	1	-	1	-	-
CMT	15	0.02	0.02	500	7.5	1	-	-	1	-
CMW	15	0.02	0.02	500	7.5	1	-	-	-	1

Note: CM, chia mucilage; C, κ-carrageenan; L, locust bean gum; T, thixogum; W, whey protein concentrate.

**Table 2:** Antioxidant properties (DPPH+ and ABTS+) and total phenolic content of gelled double emulsions and GTE, after 35 days of storage.

Gelled Double Emulsions	DPPH·+ (µmol TE mL^−1^)		ABTS·+ (µmol TE mL^−1^)		Total Phenols (µg CE mL^−1^)	
	Day 0	Day 35	Day 0	Day 35	Day 0	Day 35
CM	^b^ 1954.50 ± 11.40 ^A^	^b^ 1449.08 ± 80.29 ^B^	^b^ 17,343.51 ± 20.68 ^A^	^b^ 15,898.77 ± 20.68 ^B^	^d^ 3951.02 ± 82.05 ^A^	^e^ 3353.23 ± 2.86 ^B^
CMC	^c^ 2176.75 ± 44.54 ^A^	^b^ 1439.21 ± 5.70 ^B^	^c^ 18,704.68 ± 41.36 ^A^	^c^ 16,197.27 ± 54.71 ^B^	^d^ 4010.47 ± 2.86 ^A^	^d^ 3318.56 ± 2.86 ^B^
CML	^d^ 2333.15 ± 7.54 ^A^	^c^ 1839.26 ± 2.85 ^B^	^c^ 19,098.70 ± 20.68 ^A^	^e^ 17,689.77 ± 20.86 ^B^	^e^ 4135.97 ±5.72 ^A^	^f^ 3805.70 ± 8.58 ^B^
CMT	^c^ 2143.82 ± 18.08 ^A^	^c^ 1819.50 ± 11.40 ^B^	^b^ 17,844.99 ± 593.28 ^A^	^d^ 16,615.17 ± 230.29 ^B^	^b^ 2656.36 ± 42.09 ^A^	^b^ 2312.88 ± 2.86 ^B^
CMW	^c^ 2208.03 ± 69.14 ^A^	^b^ 1361.83 ± 22.81 ^B^	^c^ 18,609.16 ± 62.04 ^A^	^c^ 16,782.33 ± 35.82 ^B^	^c^ 3526.63 ± 5.72 ^A^	^c^ 2993.24 ± 8.58 ^B^
GTE	^a^ 1868.57 ± 12.43 ^A^	^a^ 208.82 ± 5.70 ^B^	^a^ 9164.30 ± 18.80 ^A^	^a^ 1685.45 ± 16.28 ^B^	^a^ 1360.49 ± 176.08 ^A^	^a^ 486.14 ± 24.99 ^B^

Note: CM (chia mucilage), CMC (chia mucilage/κ-carrageenan), CML (chia/locust bean gum), CMT (chia/thixogum), CMW (chia mucilage/whey protein concentrate) and GTE (green tea extract). Means within a row (A,B) and column (a--f) that do not have a common superscript letter are significantly different (*p* < 0.05).

**Table 3:** ABTS antioxidant activity (µmol TE mL^−1^) of gelled double emulsions (day 0) during the in vitro digestion process.

Gelled Double Emulsions	Initial Conditions (10 min)	Gastric Conditions (120 min)	Intestinal Conditions (240 min)
CM	^b^ 5134.72 ± 107.15 ^A^	^c^ 6353.32 ± 353.86 ^B^	^a^ 6465.80 ± 479.39 ^B^
CMC	^b^ 5265.95 ± 19.36 ^A^	^d^ 7228.21 ± 96.81 ^B^	^b^ 8365.56 ± 413.47 ^C^
CML	^a^ 1466.48 ± 36.96 ^A^	^a^ 1631.46 ± 53.39 ^A^	^c^ 11,008.98 ± 294.04 ^B^
CMT	^b^ 5059.73 ± 290.84 ^A^	^b^ 5697.15 ± 161.56 ^A^	^a^ 6565.79 ± 836.07 ^B^
CMW	^c^ 7315.70 ± 294.28 ^A^	^e^ 8090.60 ± 479.39 ^B^	^c^ 11,315.20 ± 117.78 ^C^
GTE	^d^ 8984.24 ± 586.05 ^A^	^f^ 9315.45 ± 411.19 ^A^	^d^ 16,470.80 ± 152.61 ^B^

Note: CM (chia mucilage), CMC (chia mucilage/κ-carrageenan), CML (chia mucilage/locust bean gum), CMT (chia mucilage/thixogum), CMW (chia mucilage/whey protein concentrate) and GTE (green tea extract). Means within a row (A,B,C) and column (a-f) that do not have a common superscript letter are significantly different (*p* < 0.05).
